# Genetic Moderation of the Association of β-Amyloid With Cognition and MRI Brain Structure in Alzheimer Disease

**DOI:** 10.1212/WNL.0000000000207305

**Published:** 2023-07-04

**Authors:** Philip S. Insel, Atul Kumar, Oskar Hansson, Niklas Mattsson-Carlgren, for the Alzheimer's Disease Neuroimaging Initiative

**Affiliations:** From the Clinical Memory Research Unit (P.S.I., A.K., O.H., N.M.-C.), Faculty of Medicine, Lund University, Sweden; Department of Psychiatry and Behavioral Sciences (P.S.I.), University of California, San Francisco; Memory Clinic (O.H.), Department of Neurology (N.M.-C.), Skåne University Hospital, and Wallenberg Center for Molecular Medicine (N.M.-C.), Lund University, Sweden.

## Abstract

**Background and Objectives:**

There is considerable heterogeneity in the association between increasing β-amyloid (Aβ) pathology and early cognitive dysfunction in preclinical Alzheimer disease (AD). At this stage, some individuals show no signs of cognitive dysfunction, while others show clear signs of decline. The factors explaining this heterogeneity are particularly important for understanding progression in AD but remain largely unknown. In this study, we examined an array of genetic variants that may influence the relationships among Aβ, brain structure, and cognitive performance in 2 large cohorts.

**Methods:**

In 2,953 cognitively unimpaired participants from the Anti-Amyloid Treatment in Asymptomatic Alzheimer disease (A4) study, interactions between genetic variants and 18F-Florbetapir PET standardized uptake value ratio (SUVR) to predict the Preclinical Alzheimer Cognitive Composite (PACC) were assessed. Genetic variants identified in the A4 study were evaluated in the Alzheimer Disease Neuroimaging Initiative (ADNI, N = 527) for their association with longitudinal cognition and brain atrophy in both cognitively unimpaired participants and those with mild cognitive impairment.

**Results:**

In the A4 study, 4 genetic variants significantly moderated the association between Aβ load and cognition. Minor alleles of 3 variants were associated with additional decreases in PACC scores with increasing Aβ SUVR (rs78021285, β = −2.29, SE = 0.40, *p*_FDR_ = 0.02, nearest gene *ARPP21*; rs71567499, β = −2.16, SE = 0.38, *p*_FDR_ = 0.02, nearest gene *PPARD*; and rs10974405, β = −1.68, SE = 0.29, *p*_FDR_ = 0.02, nearest gene *GLIS3*). The minor allele of rs7825645 was associated with less decrease in PACC scores with increasing Aβ SUVR (β = 0.71, SE = 0.13, *p*_FDR_ = 0.04, nearest gene *FGF20*). The genetic variant rs76366637, in linkage disequilibrium with rs78021285, was available in both the A4 and ADNI. In the A4, rs76366637 was strongly associated with reduced PACC scores with increasing Aβ SUVR (β = −1.01, SE = 0.21, *t* = −4.90, *p* < 0.001). In the ADNI, rs76366637 was associated with accelerated cognitive decline (χ^2^ = 15.3, *p* = 0.004) and atrophy over time (χ^2^ = 26.8, *p* < 0.001), with increasing Aβ SUVR.

**Discussion:**

Patterns of increased cognitive dysfunction and accelerated atrophy due to specific genetic variation may explain some of the heterogeneity in cognition in preclinical and prodromal AD. The genetic variant near *ARPP21* associated with lower cognitive scores in the A4 and accelerated cognitive decline and brain atrophy in the ADNI may help to identify those at the highest risk of accelerated progression of AD.

A key hallmark of Alzheimer disease (AD) is the accumulation of β-amyloid (Aβ) pathology in the brain, which precedes significant cognitive decline and dementia.^1^ However, there is considerable heterogeneity in the association between elevated levels of Aβ pathology and early cognitive dysfunction. Some individuals show no signs of cognitive dysfunction while harboring a considerable Aβ burden, while others show clear signs of decline.^2-6^ Identifying the factors that explain this variation in cognitive performance in individuals with elevated levels of Aβ and predicting future cognitive changes are key to understanding early changes in AD.

The extent to which genetic factors account for the variation in incipient cognitive decline in preclinical AD is largely unknown. The relationship between common AD risk variants of the *APOE* gene and both cognition and the accumulation of Aβ has been consistently shown.^7-9^ Several other genetic variants (GVs) have been identified for their association with cognition during the course of AD, including Klotho-VS^10,11^ and brain-derived neurotrophic factor.^12,13^ In addition, polygenic risk scores have been developed to aggregate genetic risk across the genome for AD and cognitive decline.^14,15^ Still, a large proportion of the variation in cognitive ability and longitudinal change remains unexplained. Preclinical AD comprises a mixture of individuals who remain cognitively stable and those who decline over time, resulting in high variance estimates and small effect sizes (ESs) of change, requiring large sample sizes and long studies to detect associations between Aβ and early cognitive change. This is particularly difficult for clinical trial design where drug effects may be modest.

In this genome-wide association study, we examined how genetic variation interacts with Aβ to predict cognitive performance in 2 large, well-characterized cohorts of nondemented older adults. Cross-sectional data from the Anti-Amyloid Treatment in Asymptomatic Alzheimer disease (A4) study^16^ were used to identify significant genetic modifiers of the association between Aβ and cognition. The large sample of cognitively unimpaired (CU) participants in the A4 study with both genetic and Aβ information make it particularly well-suited as a discovery cohort to detect mild effects in the early stages of AD, before overt cognitive impairment. Significant genetic modifiers identified in A4 were then evaluated in the Alzheimer Disease Neuroimaging Initiative^17^ (ADNI) to assess whether these associations would extend to a longitudinal setting for changes in cognition and structural MRI and in individuals with mild cognitive impairment (MCI).

## Methods

### Participants

Participants from the A4 study^18^ and ADNI^17^ were included in this study. Participants screened for the A4 study were included in this study if they completed a PET scan, completed a battery of neuropsychological testing, had available genetic data, had a Clinical Dementia Rating of 0, scored between 25 and 30 on the Mini-Mental State Examination (MMSE), and were between the ages of 65 and 85 years. Exclusion criteria for the A4 study have been previously described.^16^ In brief, A4 exclusions included taking a prescription Alzheimer medication or had a current serious or unstable illness that could interfere with the study. Note that participants without evidence of brain Aβ at screening were not randomized to treatment in the A4 study but were included in this study.

Participants from the ADNI were included if they completed an 18F-Florbetapir PET scan, had available genetic data, completed a battery of neuropsychological testing, and were either CU or had MCI at baseline.

### 18F-Florbetapir PET Imaging

In the A4 study, amyloid β PET imaging was performed using 18F-Florbetapir, acquired 50–70 minutes postinjection. Images were realigned and averaged and then spatially aligned to a standard space template. 18F-Florbetapir, sampled in a global neocortical region for Aβ, including the anterior and posterior cingulate, precuneus, medial and orbitofrontal, temporal and parietal lobes, was expressed as a standardized uptake value ratio (SUVR) with a cerebellar reference region.^16^ Aβ positivity was defined as participants with 18F-Florbetapir PET SUVR ≥1.10.^3,20,21^

Aβ PET imaging in the ADNI was performed using 18F-Florbetapir data.^22^ A global amyloid target region was calculated using 4 FreeSurfer-defined regions on each participant's corresponding structural T1 MRI (frontal, cingulate, lateral parietal, and lateral temporal) and normalized to a whole cerebellum reference region to create SUVRs. Aβ positivity was defined as participants with 18F-Florbetapir PET SUVR ≥1.10.^20^

### MRI

In the ADNI, structural brain scans were acquired using 3.0T MRI. We used a standardized protocol including T1-weighted MRI scans using a sagittal volumetric magnetization–prepared rapid gradient echo sequence.^23^ Automated volumetric measures were performed with FreeSurfer, and those examined included the amygdala, banks of the superior temporal sulcus, caudal and rostral anterior cingulate, caudal and rostral middle frontal, caudate, cuneus, entorhinal cortex, frontal pole, fusiform, hippocampus, inferior/superior parietal, inferior/middle/superior temporal, isthmus cingulate, lateral occipital, medial and lateral orbitofrontal, lingual, pallidum, paracentral, parahippocampal gyrus, pars opercularis, pars orbitalis, pars triangularis, pericalcarine, postcentral, posterior cingulate, precentral, precuneus, putamen, superior frontal, supramarginal, temporal pole, thalamus, transverse temporal, and lateral and inferior lateral ventricles. The white matter hyperintensity (WMH) volume estimation was based on a Bayesian approach to segmentation of high-resolution 3-dimensional T1 and fluid-attenuated inversion recovery sequences and has been previously described in detail (adni.loni.usc.edu).

### Cognitive Testing

A4 participants completed a neuropsychological test battery including the Preclinical Alzheimer Cognitive Composite (PACC),^24,25^ comprising the MMSE, Logical Memory Delayed Recall, Free and Cued Selective Reminding Test, and the Digit Symbol Substitution Test. Individual components were z-transformed and summed. The resulting sum was then centered on the mean and standard deviation of the Aβ-negative group.

ADNI participants completed a neuropsychological test battery including a modified version of the PACC,^24,25^ comprising the MMSE, Logical Memory Delayed Recall, Delayed Word Recall from the Alzheimer Disease Assessment Scale–Cognitive Subscale, and Trail Making Test part B (log transformed). The PACC was calculated in the same way as in the A4 study, but otherwise treated separately.

### Genetic Data

In the A4 study, genetic data underwent a quality control process limiting to variants with a call rate >95%, minor allele frequency >1%, if cryptic relatedness was identified, or if out of Hardy-Weinberg equilibrium (HWE) (*p* > 10^−6^). Imputation was performed using the European samples from the HRCr1.1.2016 reference panel (Build 37 Assembly 19). Analyses were limited to those with European ancestry.

In the ADNI, quality control was performed at the participant and genetic variation levels according to established protocols.^26^ Person-based quality control included consistency between chip-inferred and self-reported sex, call rates (1% cutoff), and intense heterozygosity. In addition, high-quality variants (autosomal biallelic variants with HWE *p* > 10^−8^, minor allele frequency ≥5% and with a call rate of >99%) were used.

### Cardiovascular Risk Factors

In the A4 study, a cardiovascular risk score based on systolic blood pressure, body mass index, smoking status, and information gathered during an initial health assessment and neurologic and physical examination was used, as previously described.^8^

### Statistical Analysis

Data from the A4 study and ADNI were analyzed separately. The A4 study was used to identify significant genetic moderators of the relationship between Aβ and the PACC. The ADNI was used to attempt to validate significant findings from A4 using longitudinal PACC change and evaluate longitudinal MRI changes.

In the A4 study, PACC scores were regressed on the interaction between 18F-Florbetapir PET SUVRs and each GV separately. PACC scores were modeled using ordinary least squares regression assuming a linear association with allele frequency. Multiple comparison adjustment for SUVR × GV interactions was performed using a false discovery rate (FDR) correction. Significant GVs were evaluated again using restricted cubic splines to capture potential nonlinearity in the relationship between SUVR and PACC scores. A single internal spline knot was placed at the median SUVR value. Wald tests were used to test for significance of the SUVR × GV interaction. Likelihood ratio tests were used to test for nonlinearity. ESs for the difference in estimated PACC scores were taken to be the difference between those with and without the minor allele at SUVR 1.35, divided by the model-estimated residual SD.

Significant GVs from the A4 study that were available in the ADNI were then evaluated for their moderating effect on the relationship between Aβ and longitudinal change in the PACC. In the ADNI, repeated measures of the PACC were regressed on the interaction between 18F-Florbetapir PET SUVRs and each GV identified in the A4 study separately, using mixed-effects regression. Models included a random intercept and slope and assumed an independent correlation structure, conditional on the random effects. Mixed-effects models directly handle differential follow-up time. PACC assessments occurring before the Aβ PET scan were excluded. Aβ SUVRs and time since baseline were parameterized using restricted cubic splines with knots placed at the median Aβ SUVR value and the median time since baseline. The interaction between GV, spline terms for Aβ SUVR, and time since baseline were tested for their association with longitudinal change in the PACC using likelihood ratio tests.

Repeated measures of structural MRI volumes were also modeled using mixed-effects regression. Regional volumes were regressed on the interaction between 18F-Florbetapir PET SUVRs and each GV, similar to models of the PACC. All models were adjusted for age, sex, and the first 5 principal components of genetic background to account for unmeasured population stratification. Models of the PACC were additionally adjusted for years of education and models of MRI volumes were additionally adjusted for intracranial volume. *p* Values for the multiple MRI regions were FDR corrected. ESs at year 3 for the difference in estimated PACC scores and significant MRI volumes were taken to be the difference between those with and without the minor allele at SUVR 1.35, divided by the model estimated residual SD.

Several sensitivity analyses were performed to evaluate the effect of covariate adjustment for MCI status (for ADNI only), *APOE* ε4 carrier status, and baseline measures of MRI outcomes that were found to be significant in the longitudinal analyses (ADNI only). We also conducted sensitivity analyses to evaluate models within CU only, MCI only, and Aβ+ only. Additional models were fit to evaluate the effect of adjusting for cardiovascular risk factors. In the ADNI, models were adjusted for log-scaled and z-transformed baseline WMH.

Associations between 18F-Florbetapir and demographics were assessed using the Spearman correlation for continuous variables and a Kruskal-Wallis test for categorical variables. *p* Values <0.05 were considered significant. All analyses were performed in R version 4.1.1.

### Standard Protocol Approvals, Registrations, and Patient Consents

Institutional review boards of all participating institutions approved this study. All participants provided informed written consent.

## Results

### A4 Cohort Characteristics

Genetic, Aβ PET, and cross-sectional cognitive data were available for 2,953 participants. Participants were 71.4 years of age on average, were 59.7% female, had an average of 16.8 years of education, were 35.7% *APOE* ε4 carriers, were 36.1% Aβ+, and had an average MMSE score of 29.0 (Table 1).

**Table 1:**
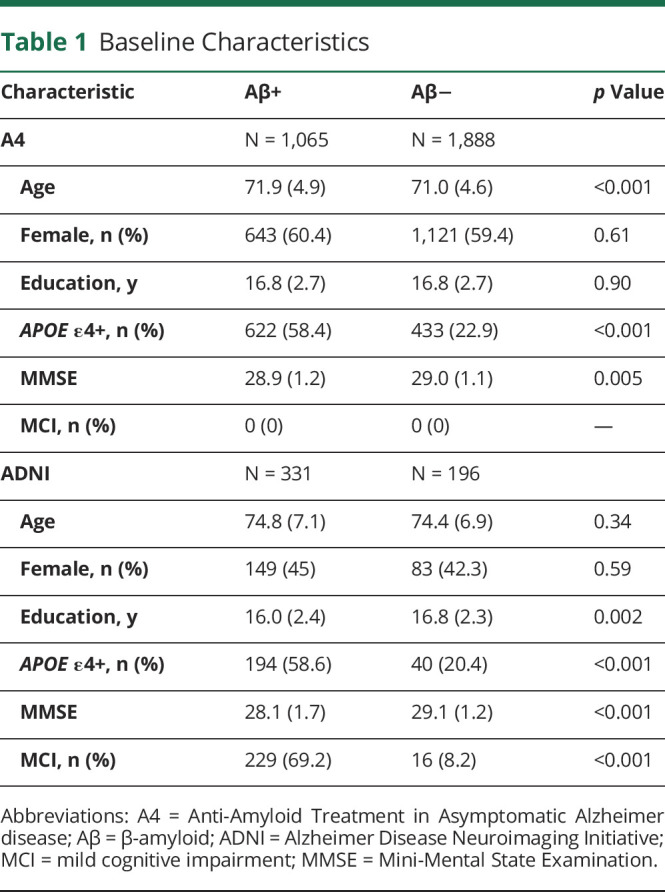
Baseline Characteristics

### Genetic Modifiers of the Relationship Between Aβ and PACC Scores

**Figure 1:**
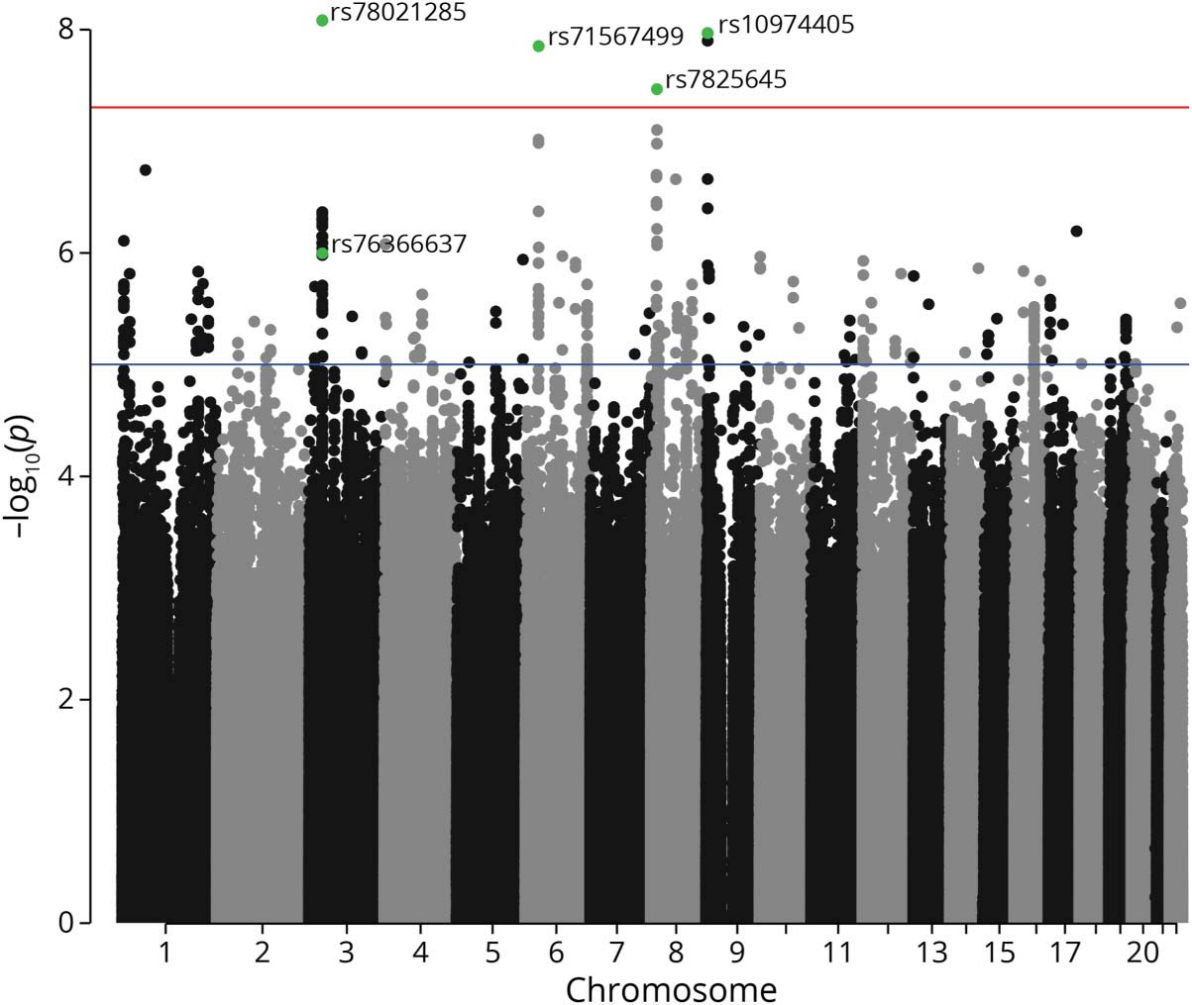
Genome-wide Significance in the A4 Genome-wide significance in the A4. A4 = Anti-Amyloid Treatment in Asymptomatic Alzheimer disease.

**Figure 2:**
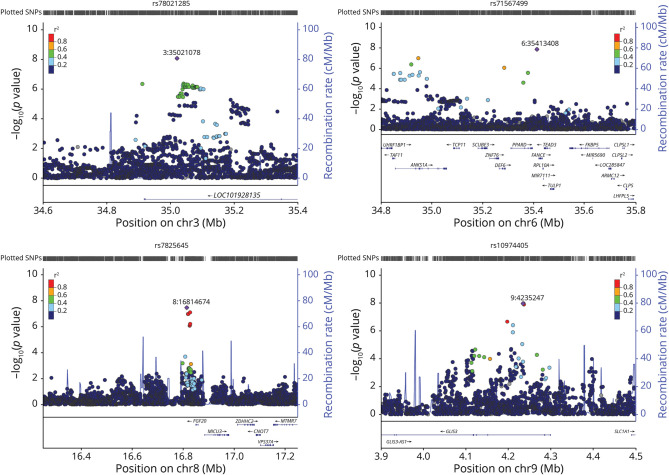
Regional Loci Regional plots of each genetic variant's locus in the A4. Selected variant shown in purple. *R*^2^ values are color coded to reflect the magnitude of linkage disequilibrium. A4 = Anti-Amyloid Treatment in Asymptomatic Alzheimer disease.

After FDR correction, 4 GVs significantly moderated the Aβ relationship with PACC performance (Figures 1 and 2). Sample characteristics of these 4 GVs are summarized in Table 2. The minor allele frequency of the 4 variants ranged from 2% to 31% (rs78021285, 2.2%; rs71567499, 2.3%; rs7825645, 30.9%; and rs10974405, 4.1%). There were 127 participants who carried the minor variant of rs78021285, there were 137 carriers of rs71567499, there were 240 carriers of rs10974405, and 290 carried 2 copies of the minor variant for rs7825645.

**Table 2:**
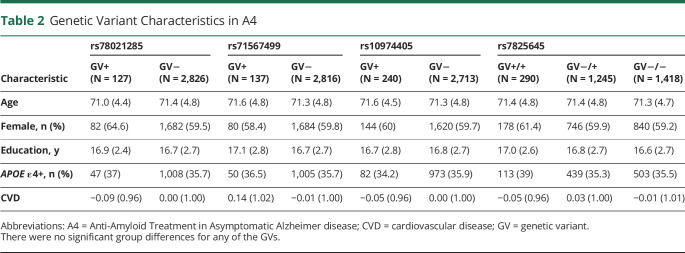
Genetic Variant Characteristics in A4

**Figure 3:**
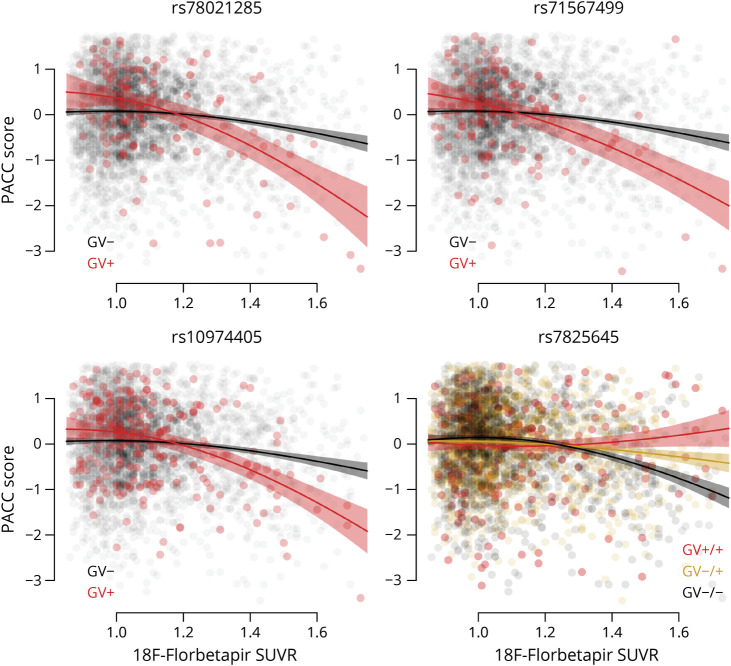
PACC Scores in A4 PACC Scores are plotted against 18F-Florbetapir SUVRs for each of the 4 variants in the A4. Separate curves are shown for participants carrying the minor allele of the genetic variant (GV+) and noncarriers (GV−). Shaded regions indicate 95% CIs. A4 = Anti-Amyloid Treatment in Asymptomatic Alzheimer disease; PACC = Preclinical Alzheimer Cognitive Composite.

The minor alleles of 3 variants were associated with an additional decrease in PACC scores with increasing Aβ SUVR (rs78021285, β = −2.29, SE = 0.40, *t* = −5.78, *p*_FDR_ = 0.02, ES at SUVR 1.35 (ES_1.35_) = −0.43; rs71567499, β = −2.16, SE = 0.38, *t* = −5.69, *p*_FDR_ = 0.02, ES_1.35_ = −0.51; rs10974405, β = −1.68, SE = 0.29, *t* = −5.73, *p*_FDR_ = 0.02, ES_1.35_ = −0.35). Trajectories of the PACC were significantly nonlinear with respect to Aβ SUVR for all GVs (rs78021285, χ^2^ = 15.78, *p* < 0.001; rs71567499, χ^2^ = 9.77, *p* = 0.002; rs10974405, χ^2^ = 13.85, *p* < 0.001; rs7825645, χ^2^ = 26.96, *p* < 0.001), as shown in Figure 3. The minor allele of rs7825645 was associated with less decrease in PACC scores with increasing Aβ SUVR (rs7825645, β = 0.71, SE = 0.13, *t* = 5.53, *p*_FDR_ = 0.04) (Figure 3).

### Sensitivity Analyses in the A4 Study

We conducted multiple sensitivity analyses to evaluate the effect of covarying for *APOE* ε4 carrier status, cardiovascular risk factors, and restricting to Aβ+ participants only. None of the sensitivity analyses changed the significance of the SUVR × GV interactions. *APOE* ε4 carrier status was not associated with PACC performance after adjusting for model covariates (β = −0.01, SE = 0.04, *p* = 0.72) and did not affect the estimates or significance of any of the significant SUVR × GV interactions. While cardiovascular risk scores were associated with reduced PACC scores (β = −0.05, SE = 0.02, *p* = 0.005), they did not affect the estimates of the SUVR × GV interactions. When restricting to Aβ+ participants only, all SUVR × GV interactions remained significant (rs78021285, χ^2^ = 16.94, *p* < 0.001; rs71567499, χ^2^ = 10.27, *p* = 0.006; rs10974405, χ^2^ = 14.92, *p* < 0.001; rs7825645, χ^2^ = 26.96, *p* < 0.001).

### Validation in the ADNI

In the ADNI, genetic, Aβ PET and cognitive data were available for 527 participants. Participants were 74.6 years of age on average, were 44% female, had an average of 16.3 years of education, were 44.4% *APOE* ε4 carriers, were 62.8% Aβ+, and had an average MMSE score of 28.4, and 46.5% had an MCI diagnosis (Table 1). Participants had 5.4 (SD = 3.4) years of follow-up and 4.5 (SD = 2.0) study visits on average.

One of the 4 GVs that was selected in the A4 study was available in the ADNI, rs10974405 on chromosome 9. Another variant available in the ADNI, rs76366637, was in linkage disequilibrium (LD) with the variant selected in the A4 study on chromosome 3 (rs78021285). In the A4 study, rs76366637 was also strongly associated with reduced PACC scores with increasing Aβ SUVR (β = −1.01, SE = 0.21, *t* = −4.90, *p* < 0.001). rs76366637 was not associated with age, sex, education, or *APOE* carrier status in either the A4 study or ADNI. rs76366637 was also not associated with cardiovascular disease in the A4 study or WMH in the ADNI. rs10974405 and rs76366637 were assessed further in the ADNI (the other 2 variants identified in the A4 study were not mapped in the ADNI). The minor allele frequency of rs10974405 was 6.6% in ADNI. The minor allele frequency of rs76366637 was 8.1% in the ADNI and 8.4% in the A4 study. In the A4 study, 15.9% (N = 471) of the participants carried the minor variant for rs76366637 and 15.2% (N = 80) in the ADNI. In the A4 study, 8.1% (N = 240) of the participants carried the minor variant for rs10974405 and 6.7% (N = 35) in the ADNI.

**Figure 4:**
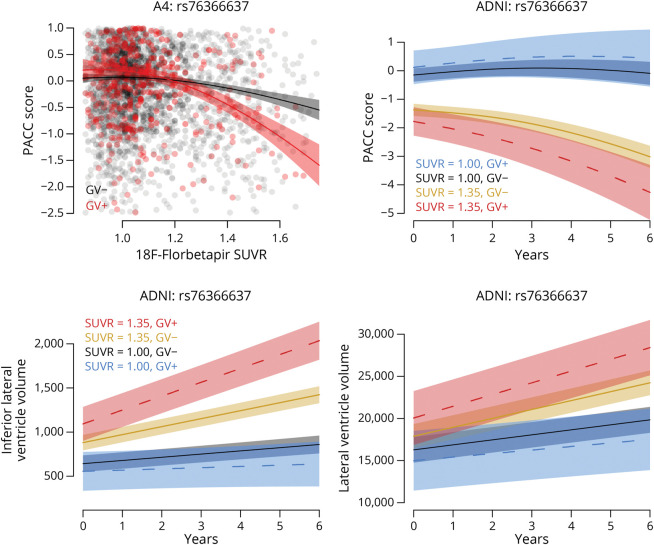
PACC Scores and Structural MRI Cross-sectional PACC scores for participants in the A4 are plotted against 18F-Florbetapir SUVRs by carrier status for the variant rs76366637 in the upper left panel. Longitudinal PACC scores for participants in the ADNI are plotted over time in the upper right panel, with estimated curves and 95% CIs for 4 groups: low amyloid GV carriers in blue, low amyloid GV noncarriers in black, high amyloid GV noncarriers in gold, and high amyloid GV carriers in red. SUVRs were modeled continuously, though specific SUVRs are used for the purpose of depiction. Similarly, in the bottom row, longitudinal ventricular volumes for ADNI participants are plotted over time with estimated curves and 95% CIs for the same 4 groups. A4 = Anti-Amyloid Treatment in Asymptomatic Alzheimer disease; ADNI = Alzheimer Disease Neuroimaging Initiative; GV = genetic variant; PACC = Preclinical Alzheimer Cognitive Composite; SUVR = standardized uptake value ratio.

In the ADNI, the minor allele of rs76366637 was associated with accelerated cognitive decline on the PACC over time with increasing Aβ SUVR (χ^2^ = 15.3, *p* = 0.004, ES at SUVR 1.35 at year 3, ES_1.35_ = −1.49, Figure 4), accelerated inferior lateral ventricular expansion over time with increasing Aβ SUVR (χ^2^ = 26.8, *p*_FDR_ < 0.001, ES_1.35_ = 3.24, Figure 4), and lateral ventricular expansion with increasing Aβ SUVR (χ^2^ = 19.7, *p*_FDR_ = 0.001, ES_1.35_ = 2.91). The minor allele of rs10974405 was not associated with an additional decline on the PACC (χ^2^ = 0.37, *p* = 0.98) or with any structural MRI measures (*p*_FDR_ > 0.09 for all).

### Sensitivity Analyses in the ADNI

While *APOE* ε4 carrier status was associated with reduced PACC scores (β = −0.38, SD = 0.13, *p* = 0.004), the effect of the interaction between rs76366637 and Aβ on longitudinal PACC change remained the same (χ^2^ = 15.5, *p* = 0.004). Similarly, the estimates for the MRI outcomes were also unchanged (inferior lateral ventricle, χ^2^ = 26.8, *p* < 0.001; lateral ventricle, χ^2^ = 19.4, *p* < 0.001).

Although WMH were associated with reduced PACC scores (β = −0.14, SE = 0.07, *p* = 0.04), model adjustment did not affect the estimate of the interaction between rs76366637 and Aβ to predict the PACC. Similarly, while baseline WMH were strongly associated with the MRI measures associated with the SUVR × GV interaction, including the inferior lateral ventricular volume expansion (β = 141.4, SE = 27.7, *p* < 0.001) and lateral ventricular volume expansion (β = 2,035, SE = 433, *p* < 0.001), adjustment for WMH did not affect the estimate or significance of the interaction between rs76366637 and Aβ with either ventricular volume outcome.

Accelerated cognitive decline and ventricular expansion remained significant when restricting to Aβ+ participants (PACC, χ^2^ = 10.47, *p* = 0.03; inferior lateral ventricles, χ^2^ = 11.1, *p* = 0.004; lateral ventricles, χ^2^ = 7.7, *p* = 0.02). When adjusting for MCI status, the minor allele of rs76366637 remained associated with accelerated cognitive decline on the PACC over time with increasing Aβ SUVR (χ^2^ = 16.0, *p* = 0.003), accelerated inferior lateral ventricular expansion over time with increasing Aβ SUVR (χ^2^ = 27.2, *p*_FDR_ < 0.001), and lateral ventricular expansion with increasing Aβ SUVR (χ^2^ = 19.9, *p*_FDR_ = 0.001).

When adjusting for baseline inferior or lateral inferior ventricular volume, estimates of the effect of the interaction between rs76366637 and Aβ on longitudinal PACC also remained the same. When considering CU participants only, the minor allele of rs76366637 remained associated with accelerated cognitive decline on the PACC over time with increasing Aβ SUVR (χ^2^ = 14.2, *p* = 0.007), accelerated inferior lateral ventricular expansion (χ^2^ = 106.3, *p*_FDR_ < 0.001), and lateral ventricular expansion (χ^2^ = 161.0, *p*_FDR_ < 0.001).

When considering MCI participants only, the minor allele of rs76366637 remained associated with accelerated cognitive decline on the PACC over time with increasing Aβ SUVR (χ^2^ = 11.1, *p* = 0.03), with accelerated inferior lateral ventricular (χ^2^ = 13.1, *p*_FDR_ = 0.03), but not with lateral ventricular expansion (χ^2^ = 9.2, *p*_FDR_ = 0.07).

## Discussion

In this study of GVs that may influence cognitive decline in preclinical and prodromal AD, 4 GVs were found to significantly moderate the relationship between Aβ and global cognitive performance in the discovery cohort (A4). In A4, 16% of the sample carried at least 1 of the 3 variants (rs78021285 on chromosome 3; rs71567499 on chromosome 6, rs10974405 on chromosome 9), which when combined with elevated levels of Aβ, resulted in significantly lower cognitive scores on average compared with individuals without these risk variants. rs76366637 (in LD with rs78021285) on chromosome 3 was associated with a lower cognitive performance in A4 and both cognitive decline and increased rates of atrophy in the ADNI with increasing levels of Aβ. rs10974405 on chromosome 9 was not associated with either cognitive decline or atrophy in the ADNI, while neither of the remaining 2 GVs (rs71567499, rs7825645) were mapped in the ADNI. Thus, the association with cognition of only one of the GVs identified in A4, estimated cross-sectionally, was also observed longitudinally in the ADNI.

This was an observational study, and though the exact molecular mechanisms underlying the association between the GVs, Aβ accumulation, and cognitive decline could not be determined, the identified variants do have several potential links to AD. On chromosome 3, rs78021285 is an intergenic variant in the pseudogene *FECHP1*, which is found downstream of the protein coding gene *ARPP21*. *ARPP21* encodes a neuronal phosphoprotein enriched in brain regions receiving dopaminergic innervation.^27^ *ARPP21* also regulates calmodulin signaling, believed to be associated with AD pathogenesis.^28,29^ On chromosome 6, rs71567499 is found in a region that is rich in genes (and several GVs in high LD with rs71567499 are located within or close to different genes, making it challenging to nominate a causative gene, Figure 2). The nearest gene to rs71567499 is *PPARD*, believed to be a potential risk factor of AD through its function and influence over plasma levels of lipids and apolipoproteins and its association with diabetes.^29,30^ On chromosome 9, rs10974405 is found near the protein-coding gene *GLIS3*, known to contain loci associated with both CSF tau and p-tau levels.^31^

The fourth variant, rs7825645, is located in an intergenic region on chromosome 8, closest to the coding gene *FGF20*, a growth factor, strongly enriched in the brain with reported roles in brain development. Risk variants of other *FGF20* genetic variation have been linked to an increased risk of Parkinson disease. The minor variant of rs7825645, carried by 10% of the sample, demonstrated the least decrease in cognitive scores compared with individuals carrying 1 of the 2 major variants, among individuals with elevated levels of Aβ.

Multiple GVs associated with cognitive resilience have recently been identified and replicated in independent cohorts. Among them, located on chromosome 8 similar to our findings, genetic variation at the *MTMR7*/*CNOT7*/*ZDHHC2*/*VPS37A* locus (rs12056505) was shown to be associated with cognitive resilience among those with elevated levels of Aβ.^32^ The protective association was not explained by differences in tau deposition or cerebrovascular disease, but possibly linked to synaptic plasticity and hippocampal-dependent learning and memory. In addition, a GV on chromosome 12 within the *IAPP*/*SLCO1A2* genes (rs73069071) was shown to modify the association between cortical Aβ deposition on AD-related cognitive impairment and temporal lobe atrophy.^33^

A4 data are limited by their cross-sectional nature. It is unknown whether the individuals carrying the GVs identified here and high levels of Aβ are declining over time or if they have stable, low cognitive scores on average. However, it seems that it is the combination of these variants and elevated Aβ that are associated with poor PACC performance, rather than the GVs alone. This suggests that PACC performance may only be reduced once Aβ levels become elevated later in life. This is supported by our partial validation, where one of the GVs in the ADNI, rs76366637, was associated with increased rates of cognitive decline over time. The ADNI has a considerably smaller sample size than the A4 and may not be fully powered to detect longitudinal associations, but the validation of rs76366637 is encouraging. In the ADNI, rs76366637 was also linked to accelerated brain atrophy in the presence of Aβ pathology, which supports that this GV or its genomic environment is relevant for an increased susceptibility to brain damage in individuals with preclinical and prodromal AD. More longitudinal and multimodal follow-up studies will be required to confirm the effects of the identified GVs. Such studies could, for example, include measures of tau pathology, for example, tau PET. We also note that the population in these analyses was restricted to non-Hispanic White participants and may not be fully representative of the population at risk of cognitive decline associated with AD. Exclusion criteria for the A4 also limited enrollment to those without health conditions that could interfere with participation in the study, potentially introducing bias.

Finding GVs that alter the relationship between AD pathology and cognitive functioning will help to identify those at the greatest risk of future decline. Determining the factors that predict accelerated decline will also help to expedite the completion of clinical trials, particularly within a CU population that requires large sample sizes, long recruitment, and long follow-up periods to demonstrate treatment efficacy.^1^

## Glossary

A4: Anti-Amyloid Treatment in Asymptomatic Alzheimer disease
Aβ: β-amyloid
AD: Alzheimer disease
ADNI: Alzheimer Disease Neuroimaging Initiative
CU: cognitively unimpaired
ES: effect size
FDR: false discovery rate
GV: genetic variant
HWE: Hardy-Weinberg equilibrium
LD: linkage disequilibrium
MCI: mild cognitive impairment
MMSE: Mini-Mental State Examination
PACC: Preclinical Alzheimer Cognitive Composite
SUVR: standardized uptake value ratio
WMH: white matter hyperintensity
